# A System of Practical and Scientific Physiognomy

**Published:** 1890-12

**Authors:** 


*A System of Practical and Scientific Physiognomy*. By MARY OLMSTED STANTON. Vols. II. Phila. and Lond.: F. A. DAVIS.

The ambition to write a book is, to-day, often satisfied by placing a few paragraphs, with a quotation or two, a preface and a dedication, between stiff boards. This has not satisfied our author. Two large octavo volumes, closely printed and profusely illustrated, seem scarcely to have fulfilled her desires; and what a queer mixture of philosophy and folly it is!

Read this: "The Kidney system creates or evolves *Conscientiousness*, *Integrity*, *Morality*. The *width* of the chin caused by the width of its bony structure causes conscientiousness as well as the strength and action of the Kidney system. A narrow, retreating chin shows that the kidneys are narrow and small; a broad, bony chin (if the eyes are well coloured) announces strong, large, or broad kidneys . . ."

Put this arrant nonsense alongside of the following: "A woman with a logical mind is as womanly in her nature as a man is manly who has an affectional nature, and who exhibits love for his wife and children, . . ." The outcome of this—the feeling of the affectional (*sic*) male reviewer towards the logical female writer—can in common courtesy scarcely be critical.

And yet the book is distinctly interesting. Few readers will be able to throw it aside without giving it something more than a perfunctory perusal.

